# The safety attitudes questionnaire – ambulatory version: psychometric properties of the Norwegian version for nursing homes

**DOI:** 10.1186/s12913-019-4244-5

**Published:** 2019-06-25

**Authors:** Gunnar Tschudi Bondevik, Dag Hofoss, Bettina Sandgathe Husebø, Ellen Catharina Tveter Deilkås

**Affiliations:** 1 Section for General Practice, Department of Global Public Health and Primary Care, University of Bergen, Bergen, Norway; 2 National Centre for Emergency Primary Health Care, NORCE Norwegian Research Centre, Bergen, Norway; 3 Lovisenberg Diaconal University College, Oslo, Norway; 4 Centre for Elderly and Nursing Home Medicine, Department of Global Public Health and Primary Care, University of Bergen, Bergen, Norway; 5 Municipality of Bergen, Bergen, Norway; 6 The Norwegian Directorate of Health, Oslo, Norway; 7 Health Services Research Unit, Akershus University Hospital, Lørenskog, Norway

**Keywords:** Adverse events, Medical errors, Nursing homes, Patient safety climate, Quality improvement, Safety attitudes questionnaire

## Abstract

**Background:**

Patient safety culture involves leader and staff interaction, routines, attitudes, practices and awareness that influence risks of adverse events in patient care. The Safety Attitudes Questionnaire (SAQ) is an instrument to measure safety attitudes among health care providers. The instrument aims to identify possible weaknesses in clinical settings and motivate quality improvement interventions leading to reductions in medical errors. The Ambulatory Version of the SAQ (SAQ-A) was developed to measure safety climate in the primary care setting. The original version of the SAQ includes six major patient safety factors: Teamwork climate, Safety climate, Job satisfaction, Perceptions of management, Working conditions, and Stress recognition. Patients in nursing homes are particularly vulnerable to adverse events. We present the psychometric properties of the Norwegian translation of the SAQ-A for the nursing home setting.

**Methods:**

The study was conducted in five nursing homes in Tønsberg, Norway, in February 2016. A total of 463 employees working more than 20% received a paper version of the translated SAQ-A adapted to the Norwegian nursing home setting and responded anonymously. Filled-in questionnaires were scanned and transferred to an SPSS file. SPSS was used to estimate Cronbach alphas, corrected item-total correlations, item-to-own and item-to-other correlations, and item-descriptive statistics. The confirmatory factor analysis was done by AMOS.

**Results:**

Of the 463 health care providers, 288 (62.2%) responded to the questionnaire. The confirmatory factor analysis showed that the total model of the six factors Teamwork climate, Safety climate, Job satisfaction, Perceptions of management, Working conditions, and Stress recognition had acceptable goodness-of-fit values in the nursing home setting.

**Conclusions:**

The results of our study indicate that the Norwegian translated version of the SAQ-A, with the confirmed six factor model, is an appropriate tool for measuring patient safety climate in the nursing home setting. Future research should study whether there is an association between patient safety climate in nursing homes and occurrence of adverse events among the patients.

**Electronic supplementary material:**

The online version of this article (10.1186/s12913-019-4244-5) contains supplementary material, which is available to authorized users.

## Background

Over the last decades, there has been an increasing interest in patient safety and learning from medical errors. Traditionally, these issues have to a large degree been addressed in hospital settings [1–7]. However, during recent years, there has been a stronger emphasis on investigating factors related to patient safety in primary care as well, as the largest volume of health care is provided outside the hospitals.

Patient safety culture involves leader and staff interaction, routines, attitudes, practices and awareness that influence risks of adverse events in patient care [8]. The concept is developed within organizational psychology, and is regarded as a group phenomenon. The safety culture in the health service near the patients is of particular interest, as a substantial variation across wards has been shown [9]. This variation is associated with risk of medical errors [10–12].

Nursing homes may be among the primary care services with the highest risks of adverse events. Nursing home patients are vulnerable due to their complex multiple diseases, cognitive impairment and unclear presentation of illnesses [13]. There is a risk of medication errors and fall injuries [14, 15]. In nursing homes there commonly are many health care providers with short or no formal training, like auxiliary nurses and untrained assistants. There might also be challenges with staffing ratios to meet the needs of the patients. In nursing homes, physicians are usually present only a few hours per week.

Compared to hospitals, the patient safety culture in nursing homes has been shown to be considerably poorer [16–18]. Therefore, nursing home patients might be at increased risk of harm related to medical errors. An association between patient safety culture in nursing homes and poor clinical outcomes has been reported [14, 15]. Nursing home administrators and managers seem to rate the safety culture in their institutions higher than do direct caregivers, who might be more aware of safety concerns [18, 19]. For this reason, the practice of leadership walkarounds has been recommended.

Assessment of patient safety climate in nursing homes provides an opportunity to identify improvement targets [20]. However, introducing safety projects may be challenging. An Ohio study showed that nearly half of the nursing staff found it difficult to make needed quality changes in nursing homes [21]. A “blame and shame” culture has also been observed in nursing homes [20, 22]. Patient safety climate assessment affords an opportunity to involve leadership in improving culture, as well as to set targets for improvement and divert resources if necessary [8].

There are several instruments to measure safety attitudes among health care professionals [1, 23–27]. These have traditionally been made for clinical settings within hospitals, like the Hospital Survey of Patient Safety Culture (HSOPSC) [25]. Over the last years, questionnaires have also been adapted to settings outside hospitals. This includes the Nursing Home Survey on Patient Safety Culture (NHSOPSC), which was developed by the Agency for Healthcare Research and Quality [18, 28]. A study describing the psychometric properties of the Norwegian NHSOPSC version has been published [29].

Another commonly used instrument is the Safety Attitudes Questionnaire (SAQ). It includes six major patient safety factors: Teamwork climate, Safety climate, Job satisfaction, Perceptions of management, Working conditions, and Stress recognition [30].

A number of studies have shown an association between SAQ scores and patient outcome [10–12, 26, 31–33]. The SAQ identifies areas with poor patient care and can motivate leaders to implement quality improvement strategies, whereby the risk of adverse events may be reduced [34–36]. The Safety Attitudes Questionnaire - Ambulatory Version (SAQ-A) has been developed and adjusted to the primary care setting [1] (Additional file 1). It provides the possibility of measuring effect of safety improvement interventions and change in safety attitudes [37]. The SAQ has also been used in nursing home settings [38, 39].

The SAQ-A has previously been adapted and validated for Norwegian General Practitioner (GP) practices and out-of-hours casualty clinics [40–42]. As the instrument has proved to be a useful tool for measuring safety climate in these parts of Norwegian primary care, we wanted to validate it in the nursing home setting as well. This will enable safety culture comparisons across sectors of the health services.

The aim of the present study was to develop a tool for measuring patient safety climate in nursing homes. We present the psychometric properties of the Norwegian translated version of the SAQ-A for nursing homes. We aimed to study whether the factor structure in this Norwegian version was the same as in the original questionnaire. Finally, we wanted to investigate whether the SAQ-A might be an appropriate instrument for measuring patient safety climate in the Norwegian nursing home setting.

## Methods

### Sample

The study was done in five nursing homes in Tønsberg, Norway. The total number of wards in the five nursing homes was 34, varying from three to 13 wards. In all, the five nursing homes cared for 366 patients, varying from 38 to 101 patients in each nursing home. There were totally 765 health care professionals working in the nursing homes. We did not include in the analyses employees that were working less than/equal to 20% (equivalent to one working day per week), or those being on leave during the study period (*n* = 302). Out of the remaining 463 employees, 288 (62.2%) answered the questionnaire (Table 1). The response rates varied between 56.9 and 72.2% across the five nursing homes.

**Table 1:** Patients, employees and response rates in five nursing homes in Tønsberg, Norway, 2016

	Patients (n)	Employees total (n)	Employees ≤ 20% & leave (n)	Employees > 20% invited (n)	Respondents (n)	Response rate (%)
Nursing home 1	46	80	26	54	39	72.2
Nursing home 2	38	65	17	48	29	60.4
Nursing home 3	92	201	51	150	95	63.3
Nursing home 4	101	215	92	123	70	56.9
Nursing home 5	89	204	116	88	55	62.5
Total	366	765	302	463	288	62.2

Patients = Total number of patients in nursing home

Employees total = Total number of employees in nursing home

Employees ≤20% & leave = Number of employees working **≤**20% in nursing home, or being on leave during the study period

Employees > 20% invited = Number of invited employees working > 20% in nursing home

Respondents = Number of employees working > 20% answering the SAQ

Response rate = Proportion of invited employees working > 20% answering the SAQ

The characteristics of the 288 respondents have previously been published in another paper [43] and are presented in Table 2.

**Table 2:** Characteristics of 288 employees in five nursing homes, Norway, responding to the SAQ-A, 2016

		Number	Percent
Profession	Registered nurse	78	30
	Nursing assistant	124	47
	Health worker	41	16
	Kitchen personnel	7	3
	Laundry personnel	3	1
	Secretary	1	0.4
	Other personnel	9	3
	Missing	25	
Gender	Female	241	94
	Male	16	6
	Missing	31	
Age	≤ 30 years	47	18
	31–40 years	44	17
	41–50 years	65	25
	51–60 years	78	30
	≥ 61 years	30	11
	Missing	24	
Work experience in total	≤ 5 years	44	17
	6–10 years	29	11
	11–20 years	60	23
	21–30 years	68	26
	31–40 years	56	21
	≥ 41 years	9	3
	Missing	22	
Work experience in nursing home	≤ 2 years	59	22
	3–5 years	60	22
	6–10 years	56	21
	11–20 years	57	21
	21–30 years	30	11
	≥ 31 years	6	2
	Missing	20	
Position job	21–40%	30	12
	41–60%	42	16
	61–80%	78	30
	≥ 81%	107	42
	Missing	31	
Norwegian native speaker	Yes	226	83
	No	47	17
	Missing	15	

Proportions (%) not including missing data

### Translation procedures

The original SAQ-A was translated according to the principles adapted from Beaton et al. [44]. The procedures with translation, back-translation and adaptation by an expert committee with clinicians and researchers are described in another paper [43]. Completing the SAQ-A takes approximately 15 min.

### Scoring

The SAQ-A has 62-items where the health care workers give their responses using a 5-point Likert scale: 1 = disagree strongly, 2 = disagree slightly, 3 = neutral, 4 = agree slightly, 5 = agree strongly. The category “Not applicable” was recoded into missing values in the data analyses. We reversed scores of negatively worded items. Factor scores were calculated by summing scores on items hypothesized to belong to each factor.

### Hypothesized factor structure

The original SAQ, developed at the University of Texas at Austin [30], described six factors: Teamwork climate, Safety climate, Working conditions, Job satisfaction, Perceptions of management and Stress recognition (Table 3). Not all the items in the original SAQ were covered by these six factors. The remaining items were included to provide information considered useful for local quality improvement interventions.

**Table 3:** The six factors and corresponding items in the original Safety Attitudes Questionnaire (SAQ) version (Sexton et al, 2006)

Teamwork climate	Nurse input is well received in this office
	In this office, it is difficult to speak up if I perceive a problem with patient care.
	Disagreements in this office are resolved appropriately (i.e., not *who* is right but *what* is best for the patient).
	I have the support I need from other personnel to care for patients.
	It is easy for personnel in this office to ask questions when there is something that they do not understand.
	The physicians and nurses here work together as a well-coordinated team.
Safety climate	I would feel safe being treated here as a patient.
	Medical errors are handled appropriately in this office.
	I receive appropriate feedback about my performance.
	In this office, it is difficult to discuss errors.
	I am encouraged by my colleagues to report any patient safety concerns I may have.
	The culture in this office makes it easy to learn from the errors of others.
	I know the proper channels to direct questions regarding patient safety in this office.
Working conditions	This office does a good job of training new personnel.
	All the necessary information for diagnostic and therapeutic decisions is routinely available to me.
	This office deals constructively with problem personnel.
	Trainees in my discipline are adequately supervised.
Job satisfaction	I like my job.
	Working in this office is like being part of a large family.
	This office is a good place to work.
	I am proud to work at this office.
	Morale in this office is high.
Perceptions of management	The management of this office supports my daily efforts.
	Office management does not knowingly compromise the safety of patients.
	The levels of staffing in this office are sufficient to handle the number of patients.
	I am provided with adequate, timely information about events in the office that might affect my work.
Stress recognition	When my workload becomes excessive, my performance is impaired.
	I am less effective at work when fatigued.
	I am more likely to make errors in tense or hostile situations.
	Fatigue impairs my performance during emergency situations (e.g. code or cardiac arrest).

For Teamwork climate in the present study, two items (Q35: It is easy for personnel in this nursing home ward to ask questions when there is something that they do not understand; and Q38: The physicians and nurses here work together as a well-coordinated team) were removed from the hypothesized factor structure model. One of these items (Q38) referred to cooperation with physicians, and was removed since physicians are more seldom present in nursing homes than in hospitals and GP practices. The other item (Q35) was removed as the need to ask questions may be perceived less prevalent in nursing home care. Based on the previously validated factor structure of the Norwegian SAQ-A [40], we hypothesized that Q18 (The levels of staffing in this nursing home ward are sufficient to handle the number of patients) should be moved from Perceptions of management to Working conditions, and that Q17 (The nursing home ward management does not knowingly compromise the safety of patients) in Perceptions of management should be exchanged with Q9 (Senior management of this nursing home ward is doing a good job).

### Data collection

The data were collected in the five nursing homes in February 2016. Paper versions of the SAQ-A were distributed by administrative contact persons to the health care workers. Filled-in questionnaires were returned anonymously in boxes. Questionnaires were scanned into an SPSS data file for analysis.

Reports with the SAQ-A results were sent to nursing home wards with at least five responders. The employees were recommended to discuss local patient safety factors, and to develop strategies for improvement in their own nursing home ward.

### Statistical analysis

To reduce loss of cases by listwise deletion of cases with missing data, imputation of missings were done by multiple regression analysis with SPSS v.24. Values were imputed for the “real missing”s (but not for those who failed to return a valid value by ticking the box “Not applicable”). For each variable with missing values imputed scores were predicted by the five answers (among the answers to the 28 questions in the confirmatory factor analysis) most strongly correlated to the variable in question.

SPSS was used to estimate the Cronbach alphas, corrected item-total correlation, item-to-other-factor correlations and item-descriptive statistics. An indicator of an item not belonging where it was hypothesized to sit is whether its removal would markedly improve the variable set’s Cronbach’s alpha, a measure of the extent to which the responses of items within a factor correlated pairwise. Cronbach alpha scores were considered good if between 0.70 and 0.90, and acceptable if above 0.60 [45]. Corrected item-total correlations were checked and compared to item-to-other-factor correlations to see if the items correlated more strongly with the factor they were hypothesized to belong to than with the other factors.

The hypothesized six factor model was tested by confirmatory factor analysis (CFA) in AMOS, using data from respondents answering all of the items, including the imputed responses. As factors reflect the correlation structure in the item responses, valid factors should reflect a thematic logic that is coherent with the purpose of the questionnaire. CFA provides goodness-of-fit indices which show how the survey responses comply with the pre-hypothesized factor model.

The following goodness-of-fit indices (indicators of how well the factor pattern in the survey responses conforms with the hypothesized factor model) were calculated: the chi-square/degrees of freedom ratio (χ^2^/d.f.), the p, the Root Mean Square Error of Approximation (RMSEA), the p_close_, the Comparative Fit Index (CFI) and the Hoelter 0.05. Acceptable goodness-of-fit values indicate that the SAQ-A measures patient safety climate by the hypothesized factors. The χ^2^/d.f. should be below 2.5 [46–48], the *p* value should exceed 0.05, the RMSEA should not exceed 0.08, the p_close_ value should exceed 0.05 [49], the CFI should exceed 0.90 [50]. The Hoelter 0.05 - an estimate of the largest sample for which a data set with these intercorrelations among the variables would confirm the model – should exceed 200 [51].

### Ethical considerations

This was a study on patient safety climate among employees in nursing homes, conducted in compliance with the Helsinki Declaration. The participants received written information about the purpose of the study, that data would be collected anonymously and treated in confidence. As this study did not involve patients, it was not regarded as a medical and health research project - and was thereby not affected by the Norwegian Health Research Act. Approval from the Committee for medical and health research ethics was therefore not needed. The study was approved by the Norwegian Social Science Data Services: the governmental agency for protecting survey research respondent privacy according to the Norwegian Personal Data Act (Ref. No. 2015/42892).

## Results

Table 4 presents median and mean scores with standard deviations for each of the 62 items, expressing the degree of agreement with the statements in the questionnaire. The proportions of missing values/not applicable at item levels are also shown in the table, and were on average 9.4%, ranging from 1.0 to 44.1%. 169 respondents answered all items in the hypothesized model. After imputation of missings by multiple regression analysis, 288 health care providers had responses to all items.

**Table 4:** Median and mean scores for the 62 items in the Safety Attitudes Questionnaire – Ambulatory Version (SAQ-A) among 288 health care providers in five nursing homes in Tønsberg, Norway, 2016

Statement	Missing/NA^a^ n (%)	Median score^b^ (range)	Mean score^b^ (SD^c^)
1. High levels of workload are common in this nursing home ward.	13 (4.5)	5 (1–5)	4.5 (0.9)
*2. I like my job.*	*3 (1.0)*	*5 (1–5)*	*4.7 (0.7)*
*3. Input from personnel is well received in this nursing home ward.*	*11 (3.8)*	*4 (1–5)*	*3.9 (1.0)*
*4. I would feel safe being treated here as a patient.*	*9 (3.1)*	*4 (1–5)*	*4.1 (1.0)*
*5. Medical errors are handled appropriately in this nursing home ward.*	*30 (10.4)*	*4 (1–5)*	*4.0 (1.1)*
*6. This nursing home ward does a good job of training new personnel.*	*11 (3.8)*	*4 (1–5)*	*3.9 (1.1)*
*7. All the necessary information for diagnostic and therapeutic decisions is routinely available to me.*	*26 (9.1)*	*4 (1–5)*	*3.8 (1.1)*
*8. Working in this nursing home ward is like being part of a large family.*	*16 (5.5)*	*4 (1–5)*	*3.7 (1.3)*
*9. Senior management of this nursing home ward is doing a good job.*	*16 (5.6)*	*4 (1–5)*	*3.8 (1.1)*
*10. The management of this nursing home ward supports my daily efforts.*	*14 (4.8)*	*4 (1–5)*	*3.9 (1.1)*
*11. I receive appropriate feedback about my performance.*	*7 (2.4)*	*4 (1–5)*	*3.7 (1.2)*
*12* ^***d***^ *. In this nursing home ward, it is difficult to discuss errors.*	*15 (5.2)*	*3 (1–5)*	*3.2 (1.3)*
13. Briefing other personnel before a procedure (e.g., wound care) is important for patient safety.	18 (6.3)	5 (1–5)	4.6 (0.8)
14. Briefings are common in this nursing home ward.	18 (6.2)	4 (1–5)	3.7 (1.1)
*15. This nursing home ward is a good place to work.*	*11 (3.8)*	*5 (1–5)*	*4.3 (0.9)*
16. Communication breakdowns which lead to delays in delivery of care are common.	32 (11.2)	3 (1–5)	2.8 (1.3)
17. The nursing home ward management does not knowingly compromise the safety of patients.	40 (13.9)	4 (1–5)	3.7 (1.3)
18. The levels of staffing in this nursing home ward are sufficient to handle the number of patients.	20 (6.9)	4 (1–5)	2.6 (1.3)
19. Decision making in this nursing home ward utilizes input from relevant personnel.	25 (8.7)	4 (1–5)	3.8 (1.0)
*20. I am encouraged by my colleagues to report any patient safety concerns I may have.*	*29 (10.1)*	*4 (1–5)*	*4.0 (1.1)*
*21. The culture in this nursing home ward makes it easy to learn from the errors of others.*	*15 (5.2)*	*4 (1–5)*	*3.8 (1.1)*
*22. This nursing home ward deals constructively with problem personnel.*	*16 (5.6)*	*4 (1–5)*	*3.5 (1.2)*
23. The medical equipment in this nursing home ward is adequate.	26 (9.0)	4 (1–5)	3.4 (1.1)
*24* ^*d*^ *. In this nursing home ward, it is difficult to speak up if I perceive a problem with patient care.*	*23 (8.0)*	*4 (1–5)*	*3.4 (1.3)*
*25. When my workload becomes excessive, my performance is impaired.*	*10 (3.4)*	*4 (1–5)*	*4.0 (1.2)*
*26. I am provided with adequate, timely information about events in the nursing home ward that might affect my work.*	*17 (5.9)*	*4 (1–5)*	*3.8 (1.1)*
27. I have seen others make errors that had the potential to harm patients.	23 (8.0)	3 (1–5)	2.7 (1.5)
*28. I know the proper channels to direct questions regarding patient safety in this nursing home ward.*	*19 (6.6)*	*5 (1–5)*	*4.1 (1.2)*
*29. I am proud to work at this nursing home ward.*	*8 (2.8)*	*5 (1–5)*	*4.3 (0.9)*
*30. Disagreements in this nursing home ward are resolved appropriately (i.e., not* who *is right but* what *is best for the patient).*	*16 (5.6)*	*4 (1–5)*	*4.0 (1.1)*
*31. I am less effective at work when fatigued.*	*10 (3.5)*	*4 (1–5)*	*4.0 (1.2)*
*32. I am more likely to make errors in tense or hostile situations.*	*15 (5.2)*	*4 (1–5)*	*3.8 (1.3)*
*33. Stress from personal problems adversely affects my performance.*	*24 (8.3)*	*4 (1–5)*	*3.3 (1.4)*
*34. I have the support I need from other personnel to care for patients.*	*16 (5.6)*	*5 (1–5)*	*4.3 (1.0)*
35. It is easy for personnel in this nursing home ward to ask questions when there is something that they do not understand.	23 (8.0)	5 (1–5)	4.3 (1.0)
36. Disruptions in the continuity of care can be detrimental to patient safety.	27 (9.4)	5 (1–5)	4.3 (1.0)
37. During emergencies, I can predict what other personnel are going to do next.	27 (9.4)	4 (1–5)	3.7 (1.0)
38. The physicians and nurses here work together as a well-coordinated team.	42 (14.6)	4 (1–5)	4.0 (1.0)
39. I am frequently unable to express disagreement with staff physicians in this nursing home ward.	92 (31.9)	3 (1–5)	2.5 (1.3)
40. Truly professional personnel can leave personal problems behind when working.	23 (8.0)	5 (1–5)	4.2 (1.1)
*41. Morale in this nursing home ward is high.*	*10 (3.5)*	*5 (1–5)*	*4.3 (1.0)*
*42. Trainees in my discipline are adequately supervised.*	*25 (8.7)*	*4 (1–5)*	*4.1 (1.0)*
43. I know the first and last names of all the personnel I worked with during my last shift.	19 (6.6)	4 (1–5)	3.9 (1.4)
44. I have made errors that had the potential to harm patients.	28 (9.7)	1 (1–5)	1.9 (1.3)
45. Attending health care providers in this nursing home ward are doing a good job.	23 (8.0)	5 (1–5)	4.5 (0.9)
46. All the personnel in this nursing home ward take responsibility for patient safety.	23 (8.0)	5 (1–5)	4.2 (1.0)
47. I feel fatigued when I have to get up in the morning and face another day on the job.	28 (9.7)	2 (1–5)	2.3 (1.4)
48. Patient safety is constantly reinforced as the priority in this nursing home ward.	22 (7.7)	4 (1–5)	4.0 (1.0)
49. I feel burned out from my work.	26 (9.1)	1 (1–5)	2.0 (1.3)
50. Important issues are well communicated at shift changes.	20 (6.9)	4 (1–5)	3.9 (1.2)
51. There is widespread adherence to clinical guidelines and evidence-based criteria in this nursing home ward.	35 (12.1)	4 (1–5)	4.1 (0.9)
52. I feel frustrated by my job.	23 (8.0)	2 (1–5)	2.4 (1.4)
53. I feel I am working too hard on my job.	23 (8.0)	3 (1–5)	2.9 (1.4)
54. Information obtained through incident reports is used to make patient care safer in this nursing home ward.	31 (10.8)	4 (1–5)	3.5 (1.3)
55. Personnel frequently disregard rules or guidelines (e.g., handwashing, wound care, etc.) that are established for this nursing home ward.	19 (6.6)	2 (1–5)	2.2 (1.4)
56. Fatigue impairs my performance during emergency situations.	31 (10.8)	2 (1–5)	2.5 (1.5)
57. Fatigue impairs my performance during routine care.	36 (12.6)	2 (1–5)	2.5 (1.5)
58. I am satisfied with the current referral process in this nursing home ward.	51 (17.7)	4 (2–5)	3.8 (1.0)
59. There is adequate and timely transfer of patient information between the nursing home physician and the general practitioner.	127 (44.1)	3 (1–5)	3.5 (1.1)
60. Medications are refilled in a timely manner.	78 (27.1)	4 (1–5)	4.1 (1.1)
61. Medications are refilled correctly.	88 (30.6)	4 (1–5)	4.1 (1.1)
62. Abnormal test results are frequently lost or overlooked.	80 (27.8)	1 (1–5)	1.9 (1.1)

^a^NA = Not applicable. Not included in calculations of mean scores

^b^Scoring: 1 = disagree strongly, 2 = disagree slightly, 3 = neutral, 4 = agree slightly, 5 = agree strongly.

^c^Standard deviation.

^**d**^Reverse-scored items.

Results based on answers from 288 health care providers working in five nursing homes in Tønsberg, Norway

Items included in confirmatory factor analyses in italics (*n* = 28)

The strongest disagreement was found in the statements “Abnormal test results are frequently lost or overlooked”, mean score (SD) 1.9 (1.1), and “I have made errors that had the potential to harm patients”, mean score (SD) 1.9 (1.3). The highest mean scores reflecting agreement were reported for the statements “I like my job”, mean score (SD) 4.7 (0.7), “Briefing other personnel before a procedure (e.g., wound care) is important for patient safety”, mean score (SD) 4.6 (0.8), “High levels of workload are common in this nursing home ward”, mean score (SD) 4.5 (0.9), and “Attending health care providers in this nursing home ward are doing a good job”, mean score (SD) 4.5 (0.9).

The confirmatory factor analysis showed that the hypothesized total model of six factors Teamwork climate, Safety climate, Job satisfaction, Perceptions of management, Working conditions, and Stress recognition fitted the data adequately. The goodness-of-fit indices for the model are presented in Table 5.

**Table 5:** Goodness-of-fit indices for the entire model among 288^a^ health care providers in five nursing homes, Tønsberg, Norway, 2016

	χ^2^/d.f.	P	RMSEA	Pclose	Hoelter 0.05	CFI
Entire model	618.3/335 = 1.846	<.001	.054	.144	176	.891

χ^2^/d.f.: should be below 2.5

P: should exceed 0.05

RMSEA: Root Mean Square Error of Approximation, should not exceed 0.08

Pclose: should exceed 0.05

Hoelter 0.05: should exceed 200

CFI: should exceed 0.90

^a^169 respondents answered all items in the hypothesized model. After imputation of missings by multiple regression analysis, 288 health care providers were included in the analysis

**Fig. 1:**
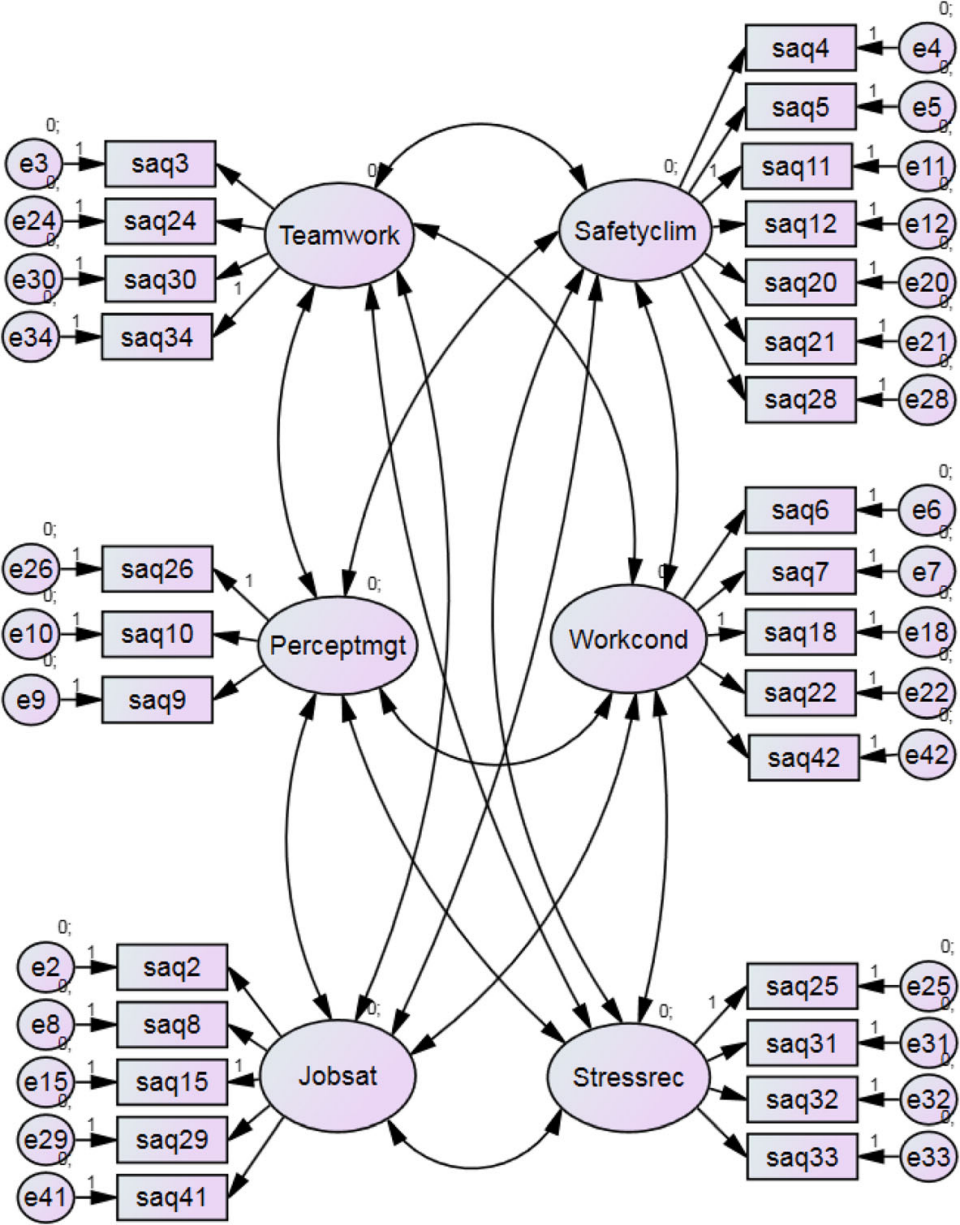
The hypothesized six-factor model, Tønsberg, Norway, 2016

The hypothesized six-factor model is presented in Figure 1.

The Cronbach’s alpha was 0.886 for the total model and ranged from 0.655 to 0.786 for the single-factor subscales: Teamwork climate, Safety climate, Working conditions, Perceptions of management, Job satisfaction and Stress recognition (Table 6).

**Table 6:** Item variation and internal consistency of the total confirmed model and of the model’s six safety factors based on data from 288^a^ respondents in five nursing homes, Tønsberg, Norway, 2016

Total model: Cronbach’s alpha: 0.886		
Teamwork climate: Cronbach’s alpha: 0.655	Mean (SD^b^)	CI-OFC^c^
3. Input from personnel is well received in this nursing home ward.	3.92 (1.01)	0.54
24^d^. In this nursing home ward, it is difficult to speak up if I perceive a problem with patient care.	3.50 (1.32)	0.33
30. Disagreements in this nursing home ward are resolved appropriately (i.e., not *who* is right but *what* is best for the patient).	4.00 (1.08)	0.55
34. I have the support I need from other personnel to care for patients.	4.30 (0.98)	0.37
Safety climate: Cronbach’s alpha: 0.738	Mean (SD^b^)	CI-OFC^c^
4. I would feel safe being treated here as a patient.	4.05 (1.03)	0.51
5. Medical errors are handled appropriately in this nursing home ward.	4.02 (1.05)	0.49
11. I receive appropriate feedback about my performance.	3.69 (1.22)	0.52
12^d^. In this nursing home ward, it is difficult to discuss errors.	3.10 (1.31)	0.36
20. I am encouraged by my colleagues to report any patient safety concerns I may have.	4.08 (1.05)	0.43
21. The culture in this nursing home ward makes it easy to learn from the errors of others.	3.79 (1.09)	0.57
28. I know the proper channels to direct questions regarding patient safety in this nursing home ward.	4.10 (1.17)	0.31
Job satisfaction: Cronbach’s alpha: 0.786	Mean (SD^b^)	CI-OFC^c^
2. I like my job.	4.70 (0.71)	0.78
8. Working in this nursing home ward is like being part of a large family.	3.67 (1.27)	0.77
15. This nursing home ward is a good place to work.	4.31 (0.89)	0.71
29. I am proud to work at this nursing home ward.	4.32 (0.93)	0.75
41. Morale in this nursing home ward is high.	4.24 (1.01)	0.79
Perceptions of management: Cronbach’s alpha: 0.713	Mean (SD^b^)	CI-OFC^c^
9. Senior management of this nursing home ward is doing a good job.	3.76 (1.13)	0.63
10. The management of this nursing home ward supports my daily efforts.	3.68 (1.09)	0.64
26. I am provided with adequate, timely information about events in the nursing home ward that might affect my work.	3.41 (1.08)	0.35
Working conditions: Cronbach’s alpha: 0.686	Mean (SD^b^)	CI-OFC^c^
6. This nursing home ward does a good job of training new personnel.	3.89 (1.10)	0.61
7. All the necessary information for diagnostic and therapeutic decisions is routinely available to me.	3.82 (1.07)	0.63
18. The levels of staffing in this nursing home ward are sufficient to handle the number of patients.	2.61 (1.31)	0.73
22. This nursing home ward deals constructively with problem personnel.	3.44 (1.22)	0.59
42. Trainees in my discipline are adequately supervised.	4.14 (0.98)	0.61
Stress recognition: Cronbach’s alpha: 0.694	Mean (SD^b^)	CI-OFC^c^
25. When my workload becomes excessive, my performance is impaired.	3.98 (1.24)	0.73
31. I am less effective at work when fatigued.	4.01 (1.21)	0.56
32. I am more likely to make errors in tense or hostile situations.	3.86 (1.24)	0.60
33. Stress from personal problems adversely affects my performance.	3.37 (1.43)	0.45

^a^169 respondents answered all items in the hypothesized model. After imputation of missings by multiple regression analysis, 288 health care providers were included in the analysis

^b^Standard deviation

^c^CI-OFC = Corrected Item-to-Own-Factor Correlation

^d^Reverse-scored items

Half (14) of the 28 items in the confirmed model correlated higher with the factor it was related to in the hypothesized factor model than with any other factor. Fourteen items correlated higher with a different factor, however, several only slightly higher. For example, Q6 correlated 0.503 to Perceptions of management and 0.497 to own factor (Working conditions).

Explained variance by factor and communality by item are presented in Table 7.

**Table 7:** Explained variance by factor, communality by item, Tønsberg, Norway, 2016

	Cumulative variance explained	Communalities
Teamwork climate	50.2%	
3. Input from personnel is well received in this nursing home ward.		.653
24. In this nursing home ward, it is difficult to speak up if I perceive a problem with patient care.		.322
30. Disagreements in this nursing home ward are resolved appropriately (i.e., not *who* is right but *what* is best for the patient).		.622
34. I have the support I need from other personnel to care for patients.		.400
Safety climate	43.4%	
4. I would feel safe being treated here as a patient.		.512
5. Medical errors are handled appropriately in this nursing home ward.		.443
11. I receive appropriate feedback about my performance.		.444
12. In this nursing home ward, it is difficult to discuss errors.		.254
20. I am encouraged by my colleagues to report any patient safety concerns I may have.		.363
21. The culture in this nursing home ward makes it easy to learn from the errors of others.		.553
28. I know the proper channels to direct questions regarding patient safety in this nursing home ward.		.471
Job satisfaction	55.6%	
2. I like my job.		.407
8. Working in this nursing home ward is like being part of a large family.		.502
15. This nursing home ward is a good place to work.		.687
29. I am proud to work at this nursing home ward.		.682
41. Morale in this nursing home ward is high.		.504
Perceptions of management	64.3%	
9. Senior management of this nursing home ward is doing a good job.		.769
10. The management of this nursing home ward supports my daily efforts.		.778
26. I am provided with adequate, timely information about events in the nursing home ward that might affect my work.		.382
Working conditions	46.6%	
6. This nursing home ward does a good job of training new personnel.		.587
7. All the necessary information for diagnostic and therapeutic decisions is routinely available to me.		.479
18. The levels of staffing in this nursing home ward are sufficient to handle the number of patients.		.169
22. This nursing home ward deals constructively with problem personnel.		.535
42. Trainees in my discipline are adequately supervised.		.559
Stress recognition	53.3%	
25. When my workload becomes excessive, my performance is impaired.		.280
31. I am less effective at work when fatigued.		.670
32. I am more likely to make errors in tense or hostile situations.		.679
33. Stress from personal problems adversely affects my performance.		.504

## Discussion

Our study showed that the hypothesized total factor model of six factors Teamwork climate, Safety climate, Job satisfaction, Perceptions of management, Working conditions, and Stress recognition had acceptable goodness-of-fit values to be used for climate measurements in the nursing home setting.

In a recent study, SAQ was shown to be a reliable tool to measure safety climate also in nursing homes in the Netherlands [38]. The authors in that study encouraged nursing homes to avoid focusing on all safety factors at the same time. Their report showed that improving one factor may have a positive influence also on other safety climate factors.

Cappelen et al. have studied the psychometric properties of the Nursing Home Survey on Patient Safety Culture (NHSOPSC) in Norwegian nursing homes [29]. This is an alternative instrument to measure safety climate. The authors identified ten factors: teamwork, staffing, compliance with procedures, training and skills, nonpunitive response to mistakes, handoffs, feedback and communication about incidents, communication openness, supervisor expectations, and management and organizational learning. Some of these factors overlap with the SAQ factors, but the NHSOPSC factors are described in more detail. Both instruments are useful tools for measuring patient safety in the Norwegian nursing home setting.

The degree of consensus amongst staff in a nursing home ward is a measure of the organizational climate’s strength. In order to describe the degree to which staff share perceptions within the same nursing home ward, we aimed to obtain a response rate of 70%. The response rate of 62.2% in our study was suboptimal. However, it was higher than the response rates in a Dutch nursing home study (53%) [38] and a study in Norwegian GP practices and out-of-hours clinics (52.2%) [40]. The large number of nursing home employees working part-time may have a higher degree of uncertainty about patient safety. This could possibly affect the willingness to participate in the study. There was no clear association between number of employees and response rates across the participating nursing homes. The actual response rates for the five nursing homes were 56.9, 60.4, 62.5, 63.3 and 72.2, respectively. This gives us the opportunity to explore the variation in organizational climate measurements across nursing home wards.

Among those responding to the questionnaire, there were moderate instances of items with missing values/not applicable, on average 9.4%. In 15 out of 62 items, the proportion of missing values/not applicable was > 10%. However, only two of these 15 items belonged to any of the safety factors. These were items Q5 (Medical errors are handled appropriately in this nursing home ward) and Q20 (I am encouraged by my colleagues to report any patient safety concerns I may have), both belonging to the factor Safety climate, and with a proportion of 10.4 and 10.1% missing values/not applicable, respectively. A high proportion of missing values/not applicable may indicate that the respondents regard the specific item as being less relevant for their nursing home setting. However, 24 out of 28 items belonging to one of the six safety factors had < 10% missing values/not applicable.

Both the size and the organization of Norwegian nursing homes vary a lot, some of them being large institutions with teaching responsibilities of health profession students, others being small with limited numbers of patients and staff. For these reasons, it is not surprising that employees may have regarded some of the 62 items as being less relevant for their particular nursing home setting. This also reflects the diversity in the included nursing homes. In addition, the included health professions have different responsibilities in nursing home care. For instance, nursing assistants are not involved in test results, and health workers have no responsibility for medication. This means that certain items are highly relevant for some of the health professions and less to others.

In our study, we chose to use the full SAQ-A version – and not the short form of SAQ. This increased the possibility of including items that staff found less appropriate – with a correspondingly larger proportion of missing responses to these items. However, this nursing home study is part of a larger patient safety climate study in different services of Norwegian primary care, and we have decided to use the full SAQ-A version in all the services.

We have to recognize that the 17% of employees who were not Norwegian native speakers may have had difficulties understanding the statements in the questionnaire. We do not know the country of origin for these respondents; language problems would probably be less among those coming from other Scandinavian countries.

As far as we know, this is the first systematic study of the psychometric properties of the SAQ-A in Norwegian nursing homes. It is a strength that the study was done in both small and large nursing homes. Comparisons of safety cultures are now possible as the SAQ-A instrument has been validated and adapted to different sectors of the primary healthcare services.

The Cronbach alphas for the total model (0.873) and for two of the six factors, Safety climate (0.700) and Job satisfaction (0.754), were considered good (≥ 0.70). For the remaining four factors, Teamwork climate (0.626), Stress recognition (0.672), Working conditions (0.673) and Perceptions of management (0.695), the Cronbach alphas were acceptable (≥0.60) [45]. These values demonstrate the internal consistency of the total model and the individual factors. The Norwegian translation of the SAQ-A adapted for nursing homes is an appropriate instrument for the study of patient safety climate in this setting.

The responses in our study tended to be skewed towards the favorable side of the scale, reflecting a positive attitude to patient safety. Still, as studies have shown that safety culture in nursing homes may be poorer than in other parts of the health services [16–18], and nursing home patients are particularly vulnerable to adverse events, it is important to address safety issues and medical errors also in this primary care setting. By measuring safety attitudes among the employees, it is possible to identify specific threats on the nursing home ward level.

We have previously studied the psychometric properties of the SAQ-A in Norwegian out-of-hours clinics and GP practices [40]. In that study, the following five factor model was shown to have acceptable goodness-of-fit values in the confirmatory factor analysis: Teamwork climate, Safety climate, Job satisfaction, Working conditions and Perceptions of management. These five factors were also confirmed in the nursing home setting. In addition, Stress recognition had acceptable goodness-of-fit values in nursing homes, but this factor was not confirmed in the GP practice or out-of-hours settings in Norway.

Despite a large degree of similarities across different health services within and between countries, important site-specific differences exist. For that reason, it is important to investigate the psychometric properties of the SAQ instrument, both in different countries and in different clinical settings in hospital and primary care.

Although authors state a correlation between patient safety culture and risk of adverse events [10–12], there are studies in which an association was not found [11, 52]. This underlines the need of further studies to investigate whether such an association applies to the nursing home setting. This was, however, beyond the scope of the present study.

The SAQ may be used to compare results before and after interventions. It also gives the possibility of comparing the relative prevalence of wards with good patient safety climate scores across institutions. This may help leaders to support health professionals in reducing the risk of medical errors. Nursing home leaders have the responsibility to encourage employees to be open about possible risks and adverse events by cultivating a “no-blame no-shame” culture. It is also important to compare safety climate across the varying services in primary and hospital care, in order to identify safety improvement strategies that can be used in different clinical settings.

## Conclusions

Our study indicates that the Norwegian translated version of the SAQ-A might be an appropriate instrument for measuring patient safety climate in the nursing home setting. Discussing the findings at ward level may facilitate interventions reducing the risk of medical errors. In future studies, possible differences in patient safety climate across nursing home wards should be investigated. Variation could imply opportunities for leaders to direct support to where improvement is most needed. Likewise, it needs to be clarified whether varying professional background, experience and age may influence attitudes to patient safety in the nursing home setting. Further research should also validate the questionnaire externally by correlating the scores on the SAQ-A domains to patient-associated outcomes in nursing homes, as this was beyond the scope of the present study.

## Additional file

Additional file 1: Safety Attitudes Questionnaire (Ambulatory Version), The University of Texas at Austin, United States, 2003. (PDF 311 kb)

## Data Availability

The datasets generated and analysed during the current study are not publicly available, as further papers will be written based on the datasets, but are available from the corresponding author on reasonable request.

## Abbreviations

CFA: Confirmatory factor analysis
GP: General Practitioner
HSOPSC: Hospital Survey of Patient Safety Culture
NHSOPSC: Nursing Home Survey on Patient Safety Culture
RMSEA: Root Mean Square Error of Approximation
SAQ: Safety Attitudes Questionnaire
SAQ-A: Safety attitudes questionnaire – ambulatory version
SPSS: Statistical package for the social sciences
χ^2^/d.f.: Chi-square/degrees of freedom ratio
