# Supplementary material for: The safety attitudes questionnaire – ambulatory version: psychometric properties of the Norwegian version for nursing homes
Source: BMC Health Serv Res. 2019 Jun 25;19:423. doi: 10.1186/s12913-019-4244-5 (PMC6593577; doi:10.1186/s12913-019-4244-5)
Supplement: Supplementary file 1 — Safety Attitudes Questionnaire (Ambulatory Version), The University of Texas at Austin, United States, 2003. (PDF 311 kb) [file 12913_2019_4244_MOESM1_ESM.pdf]

START HERE

Safety Attitudes Questionnaire (Ambulatory Version)

SERIAL #

PLEASE DO NOT WRITE IN THIS AREA

With respect to your experiences at this site, use the scale to describe the quality of collaboration and communication that you have experienced with:

Very Low / Low / Adequate / High / Very High / Not Applicable

1. Physicians Ⓐ Ⓑ Ⓒ Ⓓ Ⓔ Ⓧ
2. Registered Nurses Ⓐ Ⓑ Ⓒ Ⓓ Ⓔ Ⓧ
3. Nurse Managers Ⓐ Ⓑ Ⓒ Ⓓ Ⓔ Ⓧ
4. Residents Ⓐ Ⓑ Ⓒ Ⓓ Ⓔ Ⓧ
5. LVN Ⓐ Ⓑ Ⓒ Ⓓ Ⓔ Ⓧ
6. Radiologists Ⓐ Ⓑ Ⓒ Ⓓ Ⓔ Ⓧ
7. Medical Assistants Ⓐ Ⓑ Ⓒ Ⓓ Ⓔ Ⓧ
8. Referral Coordinators Ⓐ Ⓑ Ⓒ Ⓓ Ⓔ Ⓧ
9. Physician Office Administrator Ⓐ Ⓑ Ⓒ Ⓓ Ⓔ Ⓧ
10. Office Managers Ⓐ Ⓑ Ⓒ Ⓓ Ⓔ Ⓧ
11. Business Office Manager Ⓐ Ⓑ Ⓒ Ⓓ Ⓔ Ⓧ
12. Receptionist Ⓐ Ⓑ Ⓒ Ⓓ Ⓔ Ⓧ
13. Radiology Technicians Ⓐ Ⓑ Ⓒ Ⓓ Ⓔ Ⓧ
14. Nutritionists Ⓐ Ⓑ Ⓒ Ⓓ Ⓔ Ⓧ

MARKING INSTRUCTIONS

- **Use number 2 pencil only.**
- **Erase cleanly any mark you wish to change.**

USE A NO. 2 PENCIL ONLY

Correct Mark ⬬ **Incorrect Marks**

Please answer the following with respect to your clinical area here. Mark your answers using the following scale.

A	B	C	D	E	X
Disagree Strongly	Disagree Slightly	Neutral	Agree Slightly	Agree Strongly	Not Applicable

Disagree Strongly / Disagree Slightly / Neutral / Agree Slightly / Agree Strongly / Not Applicable

1. High levels of workload are common in this office. Ⓐ Ⓑ Ⓒ Ⓓ Ⓔ Ⓧ
2. I like my job. Ⓐ Ⓑ Ⓒ Ⓓ Ⓔ Ⓧ
3. Nurse input is well received in this office. Ⓐ Ⓑ Ⓒ Ⓓ Ⓔ Ⓧ
4. I would feel safe being treated here as a patient. Ⓐ Ⓑ Ⓒ Ⓓ Ⓔ Ⓧ
5. Medical errors* are handled appropriately in this office. Ⓐ Ⓑ Ⓒ Ⓓ Ⓔ Ⓧ
6. This office does a good job of training new personnel. Ⓐ Ⓑ Ⓒ Ⓓ Ⓔ Ⓧ
7. All the necessary information for diagnostic and therapeutic decisions is routinely available to me. Ⓐ Ⓑ Ⓒ Ⓓ Ⓔ Ⓧ
8. Working in this office is like being part of a large family. Ⓐ Ⓑ Ⓒ Ⓓ Ⓔ Ⓧ
9. Senior management of this office is doing a good job. Ⓐ Ⓑ Ⓒ Ⓓ Ⓔ Ⓧ
10. The management of this office supports my daily efforts. Ⓐ Ⓑ Ⓒ Ⓓ Ⓔ Ⓧ
11. I receive appropriate feedback about my performance. Ⓐ Ⓑ Ⓒ Ⓓ Ⓔ Ⓧ
12. In this office, it is difficult to discuss errors. Ⓐ Ⓑ Ⓒ Ⓓ Ⓔ Ⓧ
13. Briefing other personnel before a procedure (e.g., biopsy) is important for patient safety. Ⓐ Ⓑ Ⓒ Ⓓ Ⓔ Ⓧ
14. Briefings are common in this office. Ⓐ Ⓑ Ⓒ Ⓓ Ⓔ Ⓧ
15. This office is a good place to work. Ⓐ Ⓑ Ⓒ Ⓓ Ⓔ Ⓧ
16. Communication breakdowns which lead to delays in delivery of care are common. Ⓐ Ⓑ Ⓒ Ⓓ Ⓔ Ⓧ
17. Office management does not knowingly compromise the safety of patients. Ⓐ Ⓑ Ⓒ Ⓓ Ⓔ Ⓧ
18. The levels of staffing in this office are sufficient to handle the number of patients. Ⓐ Ⓑ Ⓒ Ⓓ Ⓔ Ⓧ
19. Decision making in this office utilizes input from relevant personnel. Ⓐ Ⓑ Ⓒ Ⓓ Ⓔ Ⓧ
20. I am encouraged by my colleagues to report any patient safety concerns I may have. Ⓐ Ⓑ Ⓒ Ⓓ Ⓔ Ⓧ
21. The culture in this office makes it easy to learn from the errors of others. Ⓐ Ⓑ Ⓒ Ⓓ Ⓔ Ⓧ
22. This office deals constructively with problem personnel. Ⓐ Ⓑ Ⓒ Ⓓ Ⓔ Ⓧ
23. The medical equipment in this office is adequate. Ⓐ Ⓑ Ⓒ Ⓓ Ⓔ Ⓧ
24. In this office, it is difficult to speak up if I perceive a problem with patient care. Ⓐ Ⓑ Ⓒ Ⓓ Ⓔ Ⓧ
25. When my workload becomes excessive, my performance is impaired. Ⓐ Ⓑ Ⓒ Ⓓ Ⓔ Ⓧ
26. I am provided with adequate, timely information about events in the office that might affect my work. Ⓐ Ⓑ Ⓒ Ⓓ Ⓔ Ⓧ
27. I have seen others make errors that had the potential to harm patients. Ⓐ Ⓑ Ⓒ Ⓓ Ⓔ Ⓧ
28. I know the proper channels to direct questions regarding patient safety in this office. Ⓐ Ⓑ Ⓒ Ⓓ Ⓔ Ⓧ
29. I am proud to work at this office. Ⓐ Ⓑ Ⓒ Ⓓ Ⓔ Ⓧ
30. Disagreements in this office are resolved appropriately (i.e., not *who* is right but *what* is best for the patient). Ⓐ Ⓑ Ⓒ Ⓓ Ⓔ Ⓧ
31. I am less effective at work when fatigued. Ⓐ Ⓑ Ⓒ Ⓓ Ⓔ Ⓧ
32. I am more likely to make errors in tense or hostile situations. Ⓐ Ⓑ Ⓒ Ⓓ Ⓔ Ⓧ
33. Stress from personal problems adversely affects my performance. Ⓐ Ⓑ Ⓒ Ⓓ Ⓔ Ⓧ
34. I have the support I need from other personnel to care for patients. Ⓐ Ⓑ Ⓒ Ⓓ Ⓔ Ⓧ

***Medical error is defined as any mistake in the delivery of care, by any healthcare professional, regardless of outcome.**

TURN OVER

Please answer by marking the response of your choice to the right of each item, using the letter from the scale below.

A	B	C	D	E	X
Disagree Strongly	Disagree Slightly	Neutral	Agree Slightly	Agree Strongly	Not Applicable

Item	Disagree Strongly	Disagree Slightly	Neutral	Agree Slightly	Agree Strongly	Not Applicable
35. It is easy for personnel in this office to ask questions when there is something that they do not understand.	A	B	C	D	E	X
36. Disruptions in the continuity of care can be detrimental to patient safety.	A	B	C	D	E	X
37. During emergencies, I can predict what other personnel are going to do next.	A	B	C	D	E	X
38. The physicians and nurses here work together as a well-coordinated team.	A	B	C	D	E	X
39. I am frequently unable to express disagreement with staff physicians/intensivists in this office.	A	B	C	D	E	X
40. Truly professional personnel can leave personal problems behind when working.	A	B	C	D	E	X
41. Morale in this office is high.	A	B	C	D	E	X
42. Trainees in my discipline are adequately supervised.	A	B	C	D	E	X
43. I know the first and last names of all the personnel I worked with during my last shift.	A	B	C	D	E	X
44. I have made errors that had the potential to harm patients.	A	B	C	D	E	X
45. Attending physicians/primary care providers in this office are doing a good job.	A	B	C	D	E	X
46. All the personnel in this office take responsibility for patient safety.	A	B	C	D	E	X
47. I feel fatigued when I have to get up in the morning and face another day on the job.	A	B	C	D	E	X
48. Patient safety is constantly reinforced as the priority in this office.	A	B	C	D	E	X
49. I feel burned out from my work.	A	B	C	D	E	X
50. Important issues are well communicated at shift changes.	A	B	C	D	E	X
51. There is widespread adherence to clinical guidelines and evidence-based criteria in this office.	A	B	C	D	E	X
52. I feel frustrated by my job.	A	B	C	D	E	X
53. I feel I am working too hard on my job.	A	B	C	D	E	X
54. Information obtained through incident reports is used to make patient care safer in this office.	A	B	C	D	E	X
55. Personnel frequently disregard rules or guidelines (e.g., handwashing, treatment protocols/clinical pathways, sterile field, etc.) that are established for this office.	A	B	C	D	E	X
56. Fatigue impairs my performance during emergency situations (e.g. code or cardiac arrest).	A	B	C	D	E	X
57. Fatigue impairs my performance during routine care.	A	B	C	D	E	X
58. I am satisfied with the current referral process in this office.	A	B	C	D	E	X
59. There is adequate and timely transfer of patient information between the primary care physician and the specialist.	A	B	C	D	E	X
60. Medications are refilled in a timely manner.	A	B	C	D	E	X
61. Medications are refilled correctly.	A	B	C	D	E	X
62. Abnormal test results are frequently lost or overlooked.	A	B	C	D	E	X

63. Have you completed this survey before? ○ Yes ○ No ○ Don't Know

BACKGROUND INFORMATION

Position: (mark your position)

- ○ Physicians
- ○ Registered Nurses
- ○ Nurse Managers
- ○ Residents
- ○ LVN
- ○ Radiologists
- ○ Medical Assistants
- ○ Referral Coordinators
- ○ Physician Office Administrator
- ○ Office Managers
- ○ Business Office Manager
- ○ Receptionist
- ○ Radiology Technicians
- ○ Nutritionists

How many years of experience do you have in this specialty? YEARS: 0 1 2 3 4 5 6 7 8 9 | 0 1 2 3 4 5 6 7 8 9

How many years have you worked at this office? YEARS: 0 1 2 3 4 5 6 7 8 9 | 0 1 2 3 4 5 6 7 8 9

CURRENT AGE YEARS: 0 1 2 3 4 5 6 7 8 9 | 0 1 2 3 4 5 6 7 8 9

Usual Shift

- ○ Days
- ○ Evenings
- ○ Nights
- ○ Variable Shifts

Job Status

- ○ Full-time
- ○ Part-time
- ○ Agency
- ○ Contract

Ethnic Group:

- ○ Hispanic
- ○ Black (not Hispanic)
- ○ White (not Hispanic)
- ○ Asian/Pacific Islander
- ○ Multi-ethnic
- ○ Other:___________________

Gender:

- ○ Male
- ○ Female

Optional collected as part of a cross-cultural study

Citizenship (e.g., Canadian, Filipino, USA, etc.): ______________________

Country of birth (if different): ______________________

COMMENTS: What are your top three recommendations for improving patient safety in this office?

1 ______________________

2 ______________________

3 ______________________

If more room for comments is needed, please provide your response on a separate sheet of paper.

Thank you for completing the questionnaire - Your time and participation are greatly appreciated

Copyright © 2003 by The University of Texas at Austin

Printed in U.S.A. Mark Reflex® forms by Pearson NCS MW249184-1 654321 ED99
